# Inactivation of *Pichia membranaefaciens* in Soybean Paste by Dual-Frequency and Moderate Thermosonication

**DOI:** 10.3390/foods13223600

**Published:** 2024-11-11

**Authors:** Jingya Qian, Shubei Chen, Shuhao Huo, Feng Wang, Bin Zou, Cunshan Zhou, Lei Zhang, Haile Ma

**Affiliations:** 1 School of Food and Biological Engineering, Jiangsu University, 301 Xuefu Road, Zhenjiang 212013, China; chen_shubei63@163.com (S.C.); huoshuhao@yeah.net (S.H.); fengwang@ujs.edu.cn (F.W.); binzou2009@ujs.edu.cn (B.Z.); cunshanzhou@163.com (C.Z.); zhangleifd@ujs.edu.cn (L.Z.); mhl@ujs.edu.cn (H.M.); 2 Key Laboratory for Theory and Technology of Intelligent Agricultural Machinery and Equipment, Jiangsu University, 301 Xuefu Road, Zhenjiang 212013, China; 3 Jiangsu Province and Education Ministry Cosponsored Synergistic Innovation Center of Modern Agricultural Equipment, 301 Xuefu Road, Zhenjiang 212013, China; 4 Institute of Food Physical Processing, Jiangsu University, 301 Xuefu Road, Zhenjiang 212013, China

**Keywords:** dual-frequency and moderate thermosonication, soybean paste, *Pichia membranaefaciens*, inactivation, quality analysis

## Abstract

Dual-frequency and moderate thermosonication (TS, 300 + 300 W, 20 + 40 kHz, 25~60 °C) was employed to inactivate *Pichia membranifaciens* in soybean paste. The aim was to evaluate the effect of TS on the inactivation of *P. membranaefaciens* and on the quality of soybean paste. The Weibull model fitted the survival data of *P. membranaefaciens* in thermosonicated soybean paste well and a decrease of 5 log of *P. membranaefaciens* in soybean paste was obtained at TS_50°C_, TS_55°C_, TS_60°C_, and T_65°C_ for 15.41, 7.49, 2.27, and 18.61 min. Scanning electron microscope observation revealed TS_50°C_ damaged the cell structure, leading to the leakage of intracellular contents. The physicochemical properties of soybean paste treated by TS were more retained than in paste treated by heat. The GC-MS analysis indicated that the flavor components had increased after TS treatment, especially at TS_50°C_. In conclusion, TS can inactive *P. membranaefaciens* in soybean paste without causing significant changes in its physicochemical and flavor qualities.

## 1. Introduction

Soybean paste is a semi-solid and sticky condiment with a unique flavor, which is made from the natural fermentation of soybeans and grains by *Aspergillus oryzae*, yeast, and lactobacillus [1]. In recent years, soybean paste has become one of the favorite foods in China, Japan, South Korea, Southeast Asia, and other countries due to its rich nutrients and unique flavor. It is also believed soybean paste contains polypeptides, isoflavones, saponins, polyphenols, and other bioactive substances, which can prevent liver cancer, inhibit the accumulation of fatty liver, and remove radioactive substances [2,3,4,5]. *Pichia* is commonly regarded as spoilage yeast in fermented products, and this can cause turbidity, a surface film of yeast, and an excessive estery flavor, resulting in a decline in product quality [6]. *P. membranaefaciens* is a kind of film-forming yeast that is abundant in traditional fermented paste. Moreover, *P. membranaefaciens* is able to survive in high-salinity environments. Therefore, the control of *P. membranaefaciens* is one of the most important problems in the soybean paste industry.

Thermal processing is commonly used to prolong the shelf life of paste [7]; however, high temperatures may destroy the color and nutritional quality of bean paste. Ultrasound (US) has been recognized as one of the non-thermal processing technologies with the potential to inactivate microorganisms and extend the shelf life of foods. Ultrasound is defined as an energy generated by sound waves of frequencies in the range of 18 kHz–100 MHz, which are not heard by humans [8,9]. The use of ultrasound causes a number of phenomena such as cavitation and effects accompanying cavitation [10]. Microbubbles form, grow, and collapse in cavitation, resulting in shock waves, microjets, and shear forces [11]. The strong shear force generated by US through the cavitation effect destroys the cell structure and causes the death of bacteria [12]. Ultrasound treatment at a 100% amplitude for 30 min inactivated 5 log *Escherichia coli* and 1.36 log *Saccharomyces cerevisiae* in pomegranate juice [13]. *Salmonella* spp. and *Staphylococcus aureus* had a significant reduction in chicken breast after ultrasound treatment [14]. In addition, ultrasound technology can preserve and even improve food quality. Ultrasound improved the quality of low-salt chicken meat paste added with carrageenan by reducing the size and rising the viscosity [15]. Adding sesame protein modified by high-intensity ultrasound improved the phase separation and oxidation stability of sesame paste [16]. It was also found the application of ultrasonicated Koji enhanced the physicochemical qualities and antioxidant activity of fermented soybean paste and red pepper paste [17]. However, the inactivation efficacy of US treatment alone is limited and does not ensure the microbiological safety of food products.

The combination of ultrasound and low-temperature heating (thermosonication, TS) has emerged as an alternative technology to enhance the microbial inactivation effect and minimize changes in the food sensory properties induced by thermal processing. Recently, according to the research [18], it was found that the combination of ultrasound and heat could inactivate *Bacillus subtilis* spores and a significant inactivation effect (2.43 ± 0.08 log reduction) was achieved after the combined treatment of ultrasound (20 kHz; 10 μm; 20 W/mL) and heat (80 °C) for 40 min. This technology relies on the cavitation effect of ultrasound to damage bacterial cell walls and intracellular components such as DNA, leading to cell death [19,20]. The cavitation effect refers to the phenomenon where, when an ultrasonic wave propagates in a liquid, the tiny bubbles in the liquid form, grow, and eventually collapse. The implosion of bubbles generates high temperatures (over 1000 K) and pressures (up to 500 MPa) at a micro-scale [21], which induces microbial inactivation. There are many factors related to microbial inactivation by TS, such as the bacterial species (Gram-positive and Gram-negative), bacterial growth period, medium, temperature, time, solid concentration, and ultrasonic power [22,23,24]. Garud et al. [25] have reported that compared with ultrasonic treatment (750 W; 20 kHz; 10 μm, 10 °C), a greater log reduction in the number of *E. coli* and *B. cereus* in natural sugarcane juice was obtained and less treatment time was needed under ultrasound in combination with 50 °C. The application of TS in microbial inactivation has been widely studied, such as in apple cider [26,27], milk [28,29], fruit juice [30,31,32,33], strawberry puree [34], beef slurry [35], and soy sauce [36]. However, little research has been conducted on the application of dual-frequency and moderate TS in soybean paste and the effect on the quality property.

The aims of this study were (1) to evaluate the effect of TS on the inactivation of *P. membranaefaciens* in soybean paste, (2) to model the TS inactivation kinetics of *P. membranaefaciens* in soybean paste, (3) to investigate the cell morphology after TS treatment, and (4) to examine the quality of soybean paste treated by TS.

## 2. Materials and Methods

### 2.1. Microbiology

#### 2.1.1. Preparation of Microbial Suspension

*P. membranaefaciens* (CGMCC 2.1858) was obtained from China General Microbiological Culture Collection Center. Firstly, the single colony of *P. membranaefaciens* was obtained after a growth period of two days at 28 °C on yeast-extract peptone dextrose agar (YPD). Then a loop of the single colony was inoculated into 50 mL of yeast-extract peptone dextrose, being shaken at 28 °C for two days. After incubation, 1 mL of cultured liquid was transferred into 50 mL of fresh yeast-extract peptone dextrose and incubated under the same conditions to prepare the suspension.

#### 2.1.2. Preparation of Soybean Paste Samples

Soybean paste was obtained from Foshan Haitian Flavoring Food Co., Ltd. (Foshan, China). One milliliter of *P. membranaefaciens* suspension was inoculated into 9 g of soybean paste with initial concentration of 10^5^~10^6^ CFU/mL. The inoculated paste was packed into 10 × 10 cm food-grade retort pouches that were made of a new PA-RCP material and capable of withstanding temperatures up to 121 °C.

#### 2.1.3. Microbial Enumeration

The number of *P. membranaefaciens* in soybean paste before and after processing (T and TS) was determined by spread plating onto YPD. Firstly, 2 g of the paste (T and TS) was taken out from the pouches, diluted 10-fold with PBS buffer, and mixed by a high speed vortex. Then, 1 mL of the mixed samples was diluted by using 9 mL of PBS buffer. Samples were diluted to appropriate concentration and 0.1 mL of dilution was coated on YPD plates. The plates were incubated at 28 °C for 2 days to form visible colonies. Plates with 10 to 100 colonies were used for enumeration. *P. membranaefaciens* concentration was expressed as CFU per milliliter (CFU/mL) of soybean paste.

### 2.2. Processing

#### 2.2.1. Thermal Processing

Water bath was used to treat samples and the temperatures were set at 50, 55, 60, and 65 °C.

The thermostatically controlled water bath was heated until the set temperature was reached. Samples taken in advance from the refrigerator were then put into the preheated water bath (completely immersed in water) and heated. After the treatment, the samples were immediately removed from hot water, cooled in ice water, and measured.

#### 2.2.2. Thermosonication Processing

Ultrasonic equipment (Jiangsu University, Zhenjiang, China) composed of ultrasonic generator, control panel, and ultrasonic tank was employed for thermosonication (TS) processing. Samples were processed using two ultrasonic generators with a frequency of 20 and 40 kHz, a total power of 600 W (300 W each), and amplitude of 100%. The energy input to the entire medium was pulsating (i.e., 10 s on and 3 s off). The food-grade retort pouch containing soybean paste was fixed and suspended on the iron stand with a clamp, placed in the center of the ultrasonic tank, and submerged into the water surface. The temperatures in the ultrasonic tank were monitored by peristaltic pump and set at 25, 40, 50, 55, and 60 °C, and the samples were recorded as TS_25°C_, TS_40°C_, TS_50°C_, TS_55°C_, and TS_60°C_, respectively. The treatment time depended on the TS treatment temperature, up to 20 min. After the treatment, samples were taken from the ultrasonic tank, cooled in ice water, and measured.

### 2.3. Scanning Electron Microscopy

Samples were fixed in 2.5% (*v*/*v*) glutaraldehyde overnight and washed three times with PBS buffer. Samples were subsequently dehydrated with 30%, 50%, 70%, 80%, and 90% ethanol solutions for 15 min, followed by being dehydrated twice with 100% ethyl alcohol for 15 min. The cell morphology was observed by scanning electron microscopy (GeminiSEM 300, Oberkochen, Germany).

### 2.4. Physicochemical Properties

#### 2.4.1. Color

The color coordinates (L*, a*, b*) of the samples were measured using a hand-held CR-400 colorimeter (Konica-Minolta, Osaka, Japan), calibrated before every measurement with a blank calibration tile. L* represented lightness or brightness, a* represented redness, and b* represented yellowness.

#### 2.4.2. Viscosity

The viscosity of the samples was measured by rotational rheometer (DHR-1, Waters, Milford, MA, USA). Samples were weighed and placed on the plate and balanced for 3 min and the distance between the rotor and the plate was 1 mm. The temperature of rheometer was fixed at 25 °C and the shear rate was 0.01–200 s^−1^.

#### 2.4.3. Flavor

Headspace solid-phase microextraction (HS-SPME) was employed to extract volatile compounds from soybean paste. In brief, 5 g of samples and 0.5 g of sodium chloride were mixed with 5 mL of deionized water and transferred to a glass vessel. The mixture was sealed with a polypropylene cap containing PTFE-silicon diaphragm and incubated at 55 °C for 5 min. The tip (CAR/PDMS SPME) (ANPEL Laboratory Technologies Inc., Shanghai, China) was inserted into the vessel, incubated at 50 °C for 40 min, and injected into a GC chromatograph for thermal desorption (5 min). Analysis of the volatiles was performed using Trace GC-MS system (HP6890-5973, Agilent, Santa Clara, CA, USA). Volatiles were separated by a DB-WAX chromatographic column (Agilent Technologies, Inc., Santa Clara, CA, USA) with helium as the carrier gas (flow rate of 1.0 mL/min in the splitless mode). The injector and detector temperatures were at 230 °C. The oven temperature was initially at 40 °C, raised to 130 °C at a rate of 2.5 °C/min, held for 1 min, and then further heated at 8 °C/min to 230 °C, held for 10 min. Ionization energy was 70 eV and the range of molecular weight scanned was 33–395 a.m.u. The GC-MS spectra of volatile compounds in samples were matched with NIST05 library by computer and manually. Only the results with matching degrees greater than 80% were recorded. The relative contents of compounds were calculated by area normalization method.

#### 2.4.4. The pH, Total Acid (TA), Amino Acid Nitrogen (AN), and Reducing Sugar (RS)

Firstly, 5 g of the samples was added to 50 mL of distilled water and then determined by pH meter (PHS-25, Shanghai, China). The contents of total acid (TA) and amino acid nitrogen (AN) were determined by acid–base titration method and formaldehyde value method. Reducing sugar content (RS) was determined by 3,5-dinitrosalicylic acid (DNS) colorimetry.

### 2.5. P. membranaefaciens Inactivation Model in Soybean Paste

A non-linear Weibull model was used to describe *P. membranaefaciens* inactivation data. The Weibull model (Equation (1)) [37,38] in the decimal logarithmic form is shown as follows:

(1) $$\log\frac{N}{N_{0}}=-bt^{n}$$

Here, *b* (the scale factor) is a rate parameter that is related to the velocity of microbial inactivation. *n* is the survival curve shape factor: *n* < 1 and *n* > 1 correspond to survival curves with concave-upwards (tailings) and concave-downwards (shoulders) shapes, respectively. When *n* = 1, the Weibull model becomes a model of the first-order kinetics.

### 2.6. Statistical Analysis

All data were expressed as means ± standard deviations (SDs). The ANOVA test was used to compare the average values, and Tukey post-test was performed at the level of *p* < 0.05 (SPSS22). The Origin 8.0 software was used to fit the model and the fitness was evaluated by adjusting R^2^ and model parameters.

## 3. Results and Discussion

### 3.1. Modeling the Thermosonication Inactivation Kinetics in Soybean Paste

**Figure 1:**
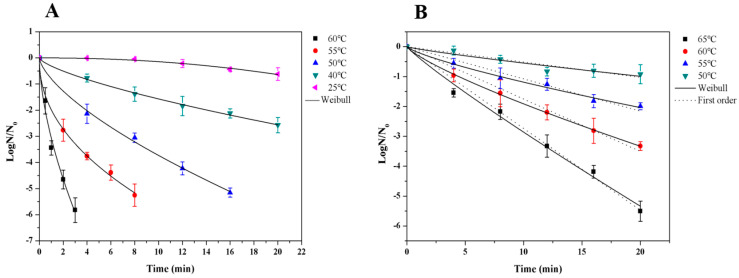
(**A**) Measured log survival data and Weibull fitted curve of *P. membranaefaciens* in soybean paste under TS treatment (20 + 40 kHz; 300 + 300 W; pulsating type: i.e., 10 s on and 3 s off). (**B**) Measured log survival data and Weibull fitted curve of *P. membranaefaciens* in soybean paste under thermal treatment.

The measured and Weibull fitted inactivation curve of *P. membranaefaciens* in soybean paste under TS and thermal treatment are shown in Figure 1. With an increase in temperature, the inactivation rate increased.

As shown in Figure 1A, the number of *P. membranaefaciens* decreased with the increase in treatment time under TS treatment and the fastest decrease occurred at TS_60°C_. When the temperature decreased, a longer time was needed to inactivate the same number of *P. membranaefaciens*. The times for inactivating about 5 log *P. membranaefaciens* in soybean paste were 15.41, 7.49, and 2.27 min at TS_50°C_, TS_55°C_, and TS_60°C_, respectively. The temperature of TS had a significant effect on the inactivation of *P. membranaefaciens*. These results were in agreement with the results obtained by Tremarin et al. [39], who reported that ultrasound at 70~95 °C inactivated more *Alicyclobacillus acidoterrestris* in apple juice than thermal treatment at 70~95 °C and that the same inactivation rate can be achieved in less than half of the time of heat treatment. The log survival rates of *P. membranaefaciens* after thermal processing for up to 20 min have been plotted in Figure 1B. With an increase in the treatment time, the number of *P. membranaefaciens* decreased. When the temperature increased, the effect on inactivation was more obvious. After treatment at 65, 60, 55, and 50 °C for 8 min, 2.2, 1.6, 1.1, and 0.4 log reductions were obtained, respectively, while 5.5, 3.3, 2, and 0.9 log reductions were achieved for 20 min.

As shown in Figure 1A, the survival curves of *P. membranaefaciens* treated by TS could not follow the first-order kinetic, with a rapid recovery at the initial stage followed by a slow inactivation stage. The Weibull model was used to fit the log survival data. The Weibull model can describe different shapes of inactivation curves and the inactivation kinetics of microorganisms exposed to thermal and non-thermal processes a show good fit with Weibull model. The model parameters estimated (*b* and *n*) are presented in Table 1. R^2^ (regression coefficient) was between 0.982 and 0.998, showing this model fitted the survival curve well. *b* values (scale factors) increased from 0.001 at 25 °C to 3.06 at 60 °C, demonstrating that this parameter was temperature-dependent. *n* values (shape factors) were between 0.46 and 0.71 (≤1) from 40 °C to 60 °C, indicating an upward concavity. However, the *n* value at 25 °C was 2.14 (>1), indicating a downward concavity. These results were in agreement with many published studies, which had reported that the Weibull model was suitable for describing the non-linear inactivation curves of different microorganisms in food such as *Alicyclobacillus acidoterrestris* [39], *Bacillus subtilis* [40], *Escherichia coli* [41], *Neosartorya fischeri* [42], and *Byssochlamys nivea* [34].

Table 1 also shows the parameters obtained by the first-order kinetic and non-linear Weibull model, which were applied to fit the inactivation data of thermal treatment. Compared with the first-order kinetic (0.917~0.987), the Weibull model showed a better fit for the inactivation curve, with a higher R^2^ (0.987~0.999) between 55 °C and 65 °C. The R^2^ value close to 1 indicated the Weibull model was adequate to describe the data. b values (scale factors) increased from 0.08 at 50 °C to 0.36 at 65 °C, indicating that this parameter was temperature-dependent. *n* values (shape factors) were between 0.78 and 0.90 (≤1), showing an upward concavity (Figure 1B).

### 3.2. Morphological Observation of P. membranaefaciens Cells

**Figure 2:**
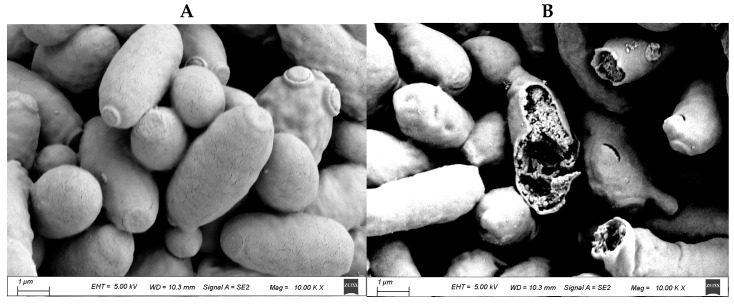
SEM micrographs of (**A**) untreated (10,000×) and (**B**) TS-treated (50 °C, 20 + 40 kHz, 300 + 300 W; pulsating type: i.e., 10 s on and 3 s off; 20 min) *P. membranaefaciens* cells (10,000×).

SEM observations of *P. membranaefaciens* cells before and after TS treatment are shown in Figure 2. The untreated *P. membranaefaciens* cells showed typical ellipsoids with smooth and intact surfaces. Conversely, cell cracks and cytoplasm leakage were observed in *P. membranaefaciens* cells treated by TS at 50 °C, indicating the cell structures were damaged. Consistent with Albanese et al. [43], yeast cell damage was observed after ultrasound.

### 3.3. Effects of Thermosonication Treatment on Physicochemical Properties of Soybean Paste

The color of soybean paste is an important sensory index to measure the quality of soybean paste. The effects of different treatments on the color of soybean paste are presented in Table 2. Thermal and TS_60°C_ treatments resulted in a significant decrease in L* values in soybean paste, which might be attributed to the Maillard browning reaction. Xu et al. [44] demonstrated that thermal and TS treatments reduced the brightness of strawberry juice and the reduction was related to oxidative browning and the Maillard reaction. All treatments reduced a* values slightly while thermal treatment decreased b* value significantly, which suggested that TS treatment was able to maintain the color of redness and yellowness in soybean paste.

**Figure 3:**
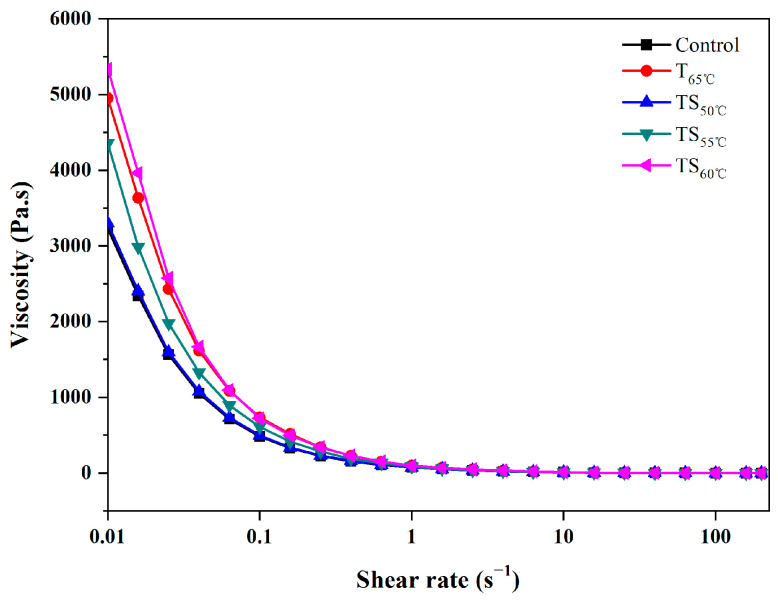
Flow curves of viscosity under different inactivation treatments.

The viscosities of soybean paste before and after different treatments are shown in Figure 3. It can be seen that the viscosity of soybean paste decreased with an increase in the shear rate. Especially when the shear rate was low, the viscosity changed rapidly, showing the characteristics of shear thinning. Therefore, all the soybean paste samples were pseudoplastic fluid. All treatments resulted in an increase in the viscosity of soybean paste, of which TS_50°C_ treatment had the smallest effect. The higher the temperature of TS treatment was, the greater the viscosity of soybean paste was.

The effects of different treatments on the pH, TA, AN, and RS of soybean paste are presented in Table 2. No significant changes (*p* > 0.05) were observed in the pH of soybean paste under the thermal and TS treatments. Conversely, a significant change (*p* < 0.05) in TA was found between TS_60°C_ and untreated samples. The composition of the substances in soybean paste was changed under thermal conditions, and the total acid content of soybean paste increased due to the production of some acidic substances. However, there were amphoteric substances in complex systems of soybean paste, which maintained the pH value while the total acid content increased. Amino acid is a polyfunctional molecule, containing at least one carboxyl group and one amino group, which can interact with a variety of flavor receptors to produce a specific flavor. The umami taste of soybean paste can be reflected by AN. As can be seen from Table 2, the content of AN in TS_50°C_-treated samples significantly increased (*p* < 0.05) compared with untreated soybean paste while the thermal-treated samples showed a lower (*p* < 0.05) AN content. The AN content can reflect the nutritional protein level in soybean paste and is considered to be the main parameter of the quality of soybean products. TS_50°C_ treatment increased the AN content, indicating an increase in the soybean paste quality. The effect of TS treatment on RS was not significant, indicating that TS treatment could efficiently maintain RS content in soybean paste. However, the RS content of samples treated by TS_50°C_ and TS_55°C_ was higher (*p* < 0.05) than that of thermal-treated samples. The high content of RS in the TS_50°C_- and TS_55°C_-treated soybean paste would provide a sweeter taste and, therefore, influence the overall sensory perception. In addition, RS can react with amino acids to form the Maillard reaction, producing substances such as melanins to enhance the color of soybean paste. An increase in the RS content may be beneficial to the taste and color of soybean paste. Similar results were obtained when TS was applied to other foods, indicating TS technology contributed to maintain or even improve the food quality [45,46,47,48,49].

### 3.4. Flavor Compound Analysis of Soybean Paste

**Figure 4:**
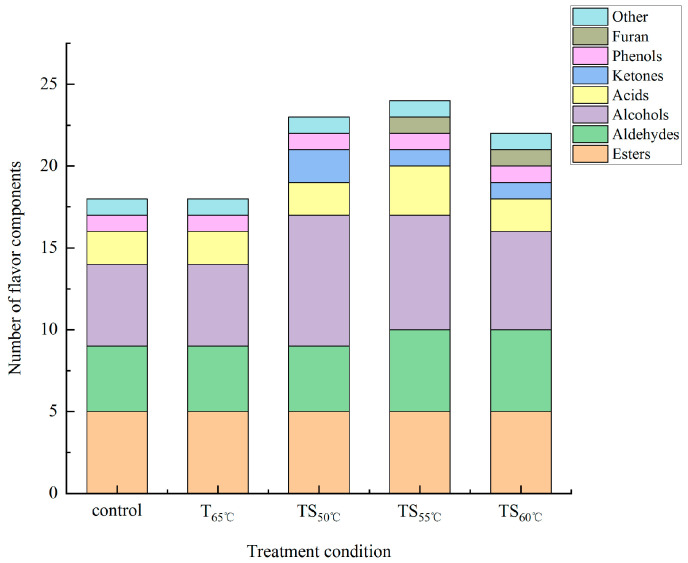
The numbers of main flavor components under different inactivation treatments.

Flavor compounds with matching degrees greater than 80% are shown in Table 3, being mainly composed of acids, alcohols, aldehydes, ketones, phenols, furans, and esters. The numbers of main flavor components in the control, thermal-treated, and TS-treated samples are shown in Figure 4. A total of 18 compounds were detected by GC-MS in control samples. The compounds in thermal-treated samples did not change compared with the untreated samples while the number of compounds in TS-treated samples increased. There were 23, 24, and 22 compounds in the samples treated by TS_50°C,_ TS_55°C,_ and TS_60°C_, respectively. New compounds formed in TS-treated samples, including 2-methylfuran, 2-butenal, 2-methyl-1-butanol, 3-octanone, 3-hydroxy-2-butanone, 2,3-butanediol, and 1,3-butanediol. TS also changed the content of the compounds in soybean paste. The changes in flavor composition in soybean paste were attributed to the effect of ultrasound. Ultrasound can induce the production of free radicals such as hydroxyl free radicals, hydrogen free radicals, and so on. These free radicals might participate in oxidation reactions and change the flavor composition in soybean paste. This result is consistent with those of other foods after ultrasound such as beer [50], wine [51] and vinegar [52].

Zhao et al. [53] reported that seven volatile compounds were identified as aroma active compounds in natural fermented soybean paste including 4-hydroxy-2(or 5)-ethyl-5(or 2)-methyl-3(2H)-furanone (soy sauce), ethyl linoleate (grease), 2,3-butanediol (burning), acetic acid (irritant sourness), fufural (yeast culture), benzene acetaldehyde (osmanthus), and pyrazine 2,6-dimethyl (cooked rice). In this study, four key compounds were also detected, including ethyl linoleate, 2,3-butanediol, acetic acid, and benzene acetaldehyde, and formed the characteristic aroma of soybean paste samples.

Esters play an important role in the aroma of soybean paste and people with lower ester thresholds are more sensitive to them [54]. Five esters were found in all samples, including ethyl acetate, ethyl benzoate, ethyl linoleate, ethyl phenyl acetate, and ethyl palmitate. Ethyl linoleic seemed to be the main ester in soybean paste owing to its highest concentration among the esters. The soybean paste also contained a high concentration of ethyl palmitate and TS (≥50 °C) decreased the content of ethyl palmitate. In addition, ethyl benzoate and ethyl phenyl acetate were detected as ethyl esters. The former has a grape-like fruit aroma with a strong flavor while the latter has an oatmeal odor with moderate and soft fragrance.

Aldehydes usually have a lower aroma threshold and higher aroma intensity. Four aromatic active aldehydes were detected in all soybean paste samples, including benzaldehyde (almond-like), benzene acetaldehyde (floral), 2-methylbutanal (malty), and 3-methylbutanal (malty). Straight-chain aliphatic aldehydes may be derived from the oxidative degradation of fatty acids while aromatic aldehydes may be derived from the Strecker degradation of amino acids. Compared with the untreated samples, the concentrations of 2-methylbutanal and 3-methylbutanal were increased under thermal and TS treatments, which may have been due to Strecker degradation between α-dicarbonyl and amino acids (isoleucine and leucine) promoted by these treatments.

Phenols are a class of compounds whose odors are easily perceived by people, and only 2-methoxy-4-vinylphenol, which provides a spicy and strong aroma in soybean paste, was detected in this study. The concentration of 2-methoxy-4-vinylphenol increased significantly after heat treatment at 65 °C. TS_55°C_ and TS_60°C_ increased the content of 2-methoxy-4-vinylphenol while TS_50°C_ decreased its content.

Alcohols give pleasant aromas and sweet flavors and five alcohols were commonly found in all the samples, including ethanol, 3-methyl-1-butanol, 1-octen-3-ol, 2-furanmethanol, and phenyl ethyl alcohol, among which ethanol and phenyl ethyl alcohol were the relatively abundant compounds. Phenyl ethyl alcohol is formed by further reduction of phenylalanine produced by the degradation of Strecker under the action of yeast. Ethanol is produced mainly by the anaerobic respiration of yeast. Compared with the untreated group, the concentration of ethanol decreased after all treatments, which may have been due to the volatility of ethanol at a certain temperature.

Some acids, furans, ketones, and nitrogen-containing compounds were also detected in soybean paste. Acetic acid was the major acid detected in the samples. Nitrogen-containing compounds are mainly formed by the thermal decomposition of proteins and amino acids and Maillard reactions of sugars with proteins or amino acids.

Overall, the aroma components of soybean paste after TS treatment increased, especially after TS_50°C_ treatment.

## 4. Conclusions

Dual-frequency and moderate thermosonication was effective to inactivate *P. membranaefaciens* in soybean paste. TS_50°C_, TS_55°C_, and TS_60°C_ significantly enhanced the inactivation effect on microorganisms compared with ultrasound and thermal treatment alone. TS_50°C_ destroyed the cell structure of *P. membranaefaciens* and caused the release of an intercellular substance, leading to cell death. The Weibull model fitted the survival data of *P. membranaefaciens* in thermosonicated soybean paste well. TS did not change the physical and chemical properties of soybean paste significantly, but increased the flavor components. TS should be considered as one of potential technologies that can replace traditional thermal processing and be applied to the fermented soybean paste industry.

## Figures and Tables

**Table 1:** Model parameters (*b* and *n*) and adjusted R^2^ in soybean paste estimated for TS and T treatments (margin of confidence intervals at 95% included).

Model	Treatment	Parameters		Adj-R^2^
		*b*	*n*	
Weibull	TS			
	60 °C	3.06 ± 0.20	0.60 ± 0.07	0.982
	55 °C	1.98 ± 0.09	0.46 ± 0.03	0.998
	50 °C	0.80 ± 0.08	0.67 ± 0.04	0.997
	40 °C	0.31 ± 0.03	0.71 ± 0.03	0.997
	25 °C	0.001 ± 7.24 × 10^−4^	2.14 ± 0.24	0.982
	T			
	65 °C	0.36 ± 0.07	0.90 ± 0.07	0.989
	60 °C	0.30 ± 0.01	0.80 ± 0.02	0.999
	55 °C	0.20 ± 0.04	0.78 ± 0.07	0.987
	50 °C	0.08 ± 0.05	0.85 ±0.22	0.907
First-order kinetic	T			
	65 °C	0.27 ± 0.01	1	0.987
	60 °C	0.18 ± 0.01	1	0.982
	55 °C	0.11 ± 0.00	1	0.968
	50 °C	0.05 ± 0.00	1	0.917

**Table 2:** Effects of TS and thermal treatments on content of color, pH, TA, AN, and RS in soybean paste (*n* = 3).

Treatment	Color			pH	TA (g/100 g)	AN (g/100 g)	RS (g/100 g)
	L*	a*	b*				
Control	35.30 ± 1.19 ^a^	8.60 ± 0.89 ^a^	12.57 ± 0.24 ^a^	5.30 ± 0.01 ^a^	1.00 ± 0.06 ^bc^	0.76 ± 0.01 ^bc^	5.00 ± 0.03 ^ab^
T_65°C_	34.39 ± 0.9 ^b^	8.36 ± 0.76 ^a^	10.94 ± 1.29 ^b^	5.30 ± 0.02 ^a^	1.06 ± 0.02 ^ab^	0.73 ± 0.02 ^c^	4.87 ± 0.04 ^c^
TS_50°C_	35.19 ± 0.36 ^ab^	8.51 ± 0.53 ^a^	13.02 ± 0.35 ^a^	5.29 ± 0.01 ^a^	0.98 ± 0.05 ^c^	0.81 ± 0.03 ^a^	5.03 ± 0.27 ^a^
TS_55°C_	35.21 ± 0.40 ^a^	8.41 ± 0.15 ^a^	12.78 ± 0.91 ^a^	5.31 ± 0.00 ^a^	1.01 ± 0.02 ^abc^	0.80 ± 0.01 ^ab^	5.07 ± 0.13 ^a^
TS_60°C_	34.37 ± 0.17 ^b^	8.27 ± 0.68 ^a^	12.64 ± 0.66 ^a^	5.30 ± 0.01 ^a^	1.08 ± 0.13 ^a^	0.77 ± 0.03 ^abc^	4.90 ± 0.07 ^bc^

Values with different letters in the same column (a–c) are significantly different (*p* < 0.05) from each other. Abbreviations: T_65°C_, thermal at 65 °C for 19 min; TS_50°C_, TS at 50 °C for 16 min; TS_55°C_, TS at 55 °C for 8 min; TS_60°C_, TS at 60 °C for 3 min.

**Table 3:** Volatile flavor compounds in soybean paste after different treatments.

Number	Retention Time	Compounds	Area%				
			Control	T_65°C_	TS_50°C_	TS_55°C_	TS_60°C_
1	9.83	2-Methylfuran				0.20	0.31
2	10.21	Ethyl Acetate	1.87	2.15	2.23	2.66	2.22
3	10.94	2-Methylbutanal	5.5	5.95	6.15	6.83	6.14
4	11.06	3-Methylbutanal	4.8	5.13	5.47	5.98	5.75
5	11.58	Ethanol	10.68	3.84	4.95	5.29	4.05
6	15.48	2-Butenal				0.39	0.36
7	23.97	2-Methyl-1-butanol				0.14	
8	24.09	3-Methyl-1-butanol			0.55	0.49	
9	25.73	3-Octanone	5.46	6.35	5.91	5.76	7.92
10	28.50	3-Hydroxy-2-butanone			0.48	0.57	0.46
11	36.09	1-Octen-3-ol			0.43		
12	36.24	Acetic acid	1.95	2.21	2.06		2.27
13	39.84	Benzaldehyde	2.37	2.87	3.67	3.82	4.30
14	40.35	2,3-Butanediol	1.73	1.80	1.58	1.67	1.65
15	41.58	1,3-Butanediol			0.63	0.48	
16	43.72	Benzene acetaldehyde			0.84	0.80	0.34
17	44.00	2-Furanmethanol	12.65	9.45	9.53	10.30	10.35
18	44.42	Ethyl benzoate	3.94	3.68	4.78	4.7	4.84
19	45.94	Oxime-, methoxy-phenyl	2.5	2.84	2.62	2.86	2.6
20	46.57	Ethyl linoleate	1.59	0.93	0.23	0.96	2.01
21	47.06	Ethyl phenyl acetate	6.48	7.73	7.80	8.37	7.95
22	49.38	Phenyl ethyl alcohol	1.56	1.38	1.41	1.71	1.8
23	49.83	2-Ethylhexanoic acid	7.36	9.62	7.12	7.04	8.32
24	54.08	2-Methoxy-4-vinylphenol	2.81	2.27	2.53	2.31	1.43
25	54.78	Ethyl palmitate	7.38	6.74	7.45	4.87	5.18

## Data Availability

The original contributions presented in the study have been included in the article; further inquiries can be directed to the corresponding author.
